# Intranasal oxytocin reduces provoked symptoms in female patients with posttraumatic stress disorder despite exerting sympathomimetic and positive chronotropic effects in a randomized controlled trial

**DOI:** 10.1186/s12916-017-0801-0

**Published:** 2017-02-17

**Authors:** M. Sack, D. Spieler, L. Wizelman, G. Epple, J. Stich, M. Zaba, U. Schmidt

**Affiliations:** 1 Technische Universität München, Klinikum rechts der Isar, Department of Psychosomatic Medicine and Psychotherapy, Langerstr. 3, 81675 München, Germany; 2 Max Planck Institute of Psychiatry, Department of Clinical Research, RG Molecular Psychotraumatology & Trauma Outpatient Clinic, Kraepelinstrasse 10, 80804 München, Germany

**Keywords:** Oxytocin, PTSD, Randomized controlled trial (RCT), Script-driven trauma imagery, Symptom provocation study, Intranasal pharmacotherapy, Psychophysiology, Trier Social Stress Test (TSST)

## Abstract

**Background:**

Posttraumatic stress disorder (PTSD) is a severe psychiatric disease accompanied by neuroendocrine changes such as adrenergic overdrive and hence an elevated cardiovascular morbidity. Current pharmacotherapeutic options for PTSD are less than suboptimal, necessitating the development of PTSD-specific drugs. Although the neuropeptide oxytocin has been repeatedly suggested to be effective in PTSD treatment, there are, to our knowledge, only three studies that have assessed its efficacy on the intensity of PTSD symptoms in PTSD patients – among them one symptom provocation study in male veterans.

**Methods:**

To evaluate for the first time how oxytocin influences the intensity of provoked PTSD symptoms and, furthermore, cardiac control in female PTSD patients, we assessed their psychic and cardiac response to trauma-script exposure with and without oxytocin pretreatment in a double-blind randomized placebo-controlled study. We used a within-subject design to study 35 female PTSD patients who received oxytocin and placebo in a 2-week interval. Furthermore, we performed a small pilot study to get an idea of the relation of the stress-modulated endogenous oxytocin levels and heart rate - we correlated oxytocin serum levels with the heart rate of 10 healthy individuals before and after exposure to the Trier Social Stress Test (TSST).

**Results:**

Intranasal oxytocin treatment was followed by a reduction of provoked total PTSD symptoms, in particular of avoidance, and by an elevation in baseline and maximum heart rate together with a drop in the pre-ejection period, a marker for sympathetic cardiac control. Furthermore, we found a positive correlation between endogenous oxytocin levels and heart rate both before and after TSST challenge in healthy control subjects.

**Conclusions:**

This study provides the first evidence that oxytocin treatment reduces the intensity of provoked PTSD symptoms in female PTSD patients. The small size of both samples and the heterogeneity of the patient sample restrict the generalizability of our findings. Future studies have to explore the gender dependency and the tolerability of the oxytocin-mediated increase in heart rate.

This randomized controlled trial was retrospectively registered at the German Trials Register (DRKS00009399) on the 02 October 2015.

**Electronic supplementary material:**

The online version of this article (doi:10.1186/s12916-017-0801-0) contains supplementary material, which is available to authorized users.

## Background

For decades, oxytocin has been used for the induction of labor and prevention of postpartum hemorrhage [1]. In recent years there has been increasing evidence that this nonapeptide, which is synthesized in the hypothalamus, might also be suitable for the treatment of psychiatric disorders such as schizophrenia, autism, major depression, anxiety disorders [2] and post-traumatic stress disorder (PTSD) [3]. In response to a variety of stimuli such as suckling, parturition and stress, oxytocin is released from the posterior pituitary into the bloodstream and transported to its effector organs such as the heart, the kidney and the brain [4]. Oxytocin acts through the oxytocin receptor (OTX) [4], which is expressed for instance in the amygdala and the anterior cingulate cortex [5], two brain regions known to be involved in the pathobiology of stress-related psychiatric diseases such as PTSD [3]. There is an unmet need for the development of drugs specifically tackling the core symptoms of this trauma spectrum disorder [6], which are hyperarousal, aversive re-experiencing, emotional numbing and avoidance anxiety, because 20–30% of PTSD patients do not respond at all to treatment with the current gold standard of PTSD drug therapy, the serotonin re-uptake inhibitors [7]. Among other neuropeptides such as neuropeptide S (NPS) [8] and neuropeptide Y (NPY) [8, 9], oxytocin has been repeatedly suggested to be effective in PTSD treatment [3, 9].

In 1993, Pitman and colleagues were the first to test the efficacy of oxytocin versus placebo in PTSD [10]. They applied either 20 IU oxytocin, vasopressin or placebo to male PTSD combat veterans before their exposure to various tasks (inter alia to combat audiovisual stimuli) and compared the heart rate (HR), electromyography, skin conductance and psychological responses between the three groups [10]. The only differences they noted were “a significantly higher baseline SC [skin conductance] level and a trend toward a higher baseline EMG [electromyographic]” response in the oxytocin group. Seventeen years later, another study, published hitherto as a poster abstract only, revealed more promising results because the authors reported a significant effect of a single dose of 24 IU oxytocin on non-provoked PTSD symptoms, in particular on the intensity of recurrent thoughts about the traumatic event and on the desire for social interaction [11, 12]. Accordingly, Olff and colleagues reported that oxytocin treatment decreased amygdalar reactivity toward emotional faces in PTSD patients [13] and Acheson and colleagues demonstrated that 24 IU of intranasal oxytocin facilitated fear extinction in healthy human subjects [14]. Currently, the efficacy of oxytocin on secondary prevention of PTSD is evaluated in a double-blind randomized placebo-controlled trial [15]. Intranasal treatment with 40 IU oxytocin was found to intensify trauma-script-induced re-experiencing symptoms in recently traumatized healthy subjects [16] but to attenuate stress reactivity, including the cortisol response, in patients with borderline personality disorder [17], a psychiatric disease known to have a high comorbidity and symptom overlap with PTSD [18]. In addition, two other studies have demonstrated that intranasal oxytocin reduces stress-induced cortisol levels [19, 20], thereby emphasizing the interrelationship of the oxytocin system and the hypothalamic-pituitary-adrenal axis, which, in turn, has been repeatedly shown to play a central role in PTSD [6, 21].

There are, to the best of our knowledge, only two studies thus far that have analyzed the efficacy of oxytocin on PTSD symptom intensity in PTSD patients, among them one symptom provocation study that was performed in a cohort of male veterans [10]. This motivated us to perform the first study analyzing the efficacy of oxytocin on provoked PTSD symptoms in female PTSD patients. Testing a female PTSD patient cohort is of particular relevance because gender differences in the effects of oxytocin have been repeatedly reported [22, 23] and because PTSD is more prevalent in women. Besides analyzing the influence of intranasal oxytocin on PTSD symptoms, we put particular emphasis on evaluating its effects on cardiac control, because, apart from oxytocin’s undisputed role in cardiovascular regulation [24], its effects on HR remain unclear: on the one hand, oxytocin has been reported to reduce HR [24]; on the other hand, there are numerous publications reporting that oxytocin treatment, especially oxytocin bolus application, can lead to tachycardia [25, 26]. Accordingly, there are studies showing that oxytocin increases HR in mice [27, 28], rats [29] and dogs [30]. In contrast, Pitman and colleagues found no changes in HR in male PTSD veterans, neither at baseline nor in response to combat audiovisual stimulus [10]. In consequence of these discrepancies, Novartis reports that “cardiovascular changes including tachycardia and bradycardia can be common” (p.1115) in response to oxytocin treatment [31]. There is one study that nicely demonstrated the influence of individual psychological factors on oxytocin-mediated cardiac control by showing that higher levels of loneliness were associated with reduced parasympathetic cardiac reactivity to intranasal oxytocin [32]. In the latter study, oxytocin significantly increased overall autonomic cardiac control because it elevated high frequency heart rate variability (HRV) and decreased the pre-ejection period (PEP), a well-known indicator of sympathetic cardiac control. Because PTSD dramatically increases the risk for cardiovascular mortality [33], it is of utmost importance to study the influence of potential PTSD drugs on cardiac parameters. In the study presented here, we analyzed, in addition to assessing the psychological response, the respiration rate and several cardiac parameters, namely HR, HRV and PEP, not only at baseline but also after symptom provocation.

## Methods

### Demographic and clinical characteristics of the PTSD patient cohort (trauma-script challenge experiment)

Between November 2010 and December 2012, we recruited 35 adult female Caucasian patients with full PTSD from consecutive outpatients attending the Clinic for Psychosomatic Medicine and Psychotherapy of the Technical University of Munich (TUM). At the time of analysis, patients had to fulfill the diagnostic criteria for full PTSD syndrome according to the Diagnostic and Statistical Manual of Mental Disorders, 4th edition (DSM-IV), had to be between 18 and 70 years old, and had to have sufficient knowledge of the German language to complete the questionnaires. Patients treated with beta-adrenoreceptor antagonists or other cardiovascular active drugs were excluded from the study. Additional exclusion criteria were lifetime history of psychosis, bipolar disorder, active substance abuse or suicidal ideation.

### Demographic and clinical characteristics of the control subject cohort (Trier Social Stress Test experiment)

The 10 healthy adult Caucasian women assessed here for their endogenous oxytocin plasma levels, cortisol serum levels, HR and subjective stress response constitute a subsample of a previously published cohort [21] that was subjected to the Trier Social Stress Test (TSST). Thus, this small cohort of healthy subjects was not included in the randomized clinical trial (RCT) but served as an additional experiment. They were recruited in the Trauma Outpatient Clinic of the Max Planck Institute of Psychiatry in Munich by advertisement and were free from trauma history, from any medication and from any type of psychopathology in their lifetime as detailed previously [21]. As published [21], participants of this TSST study had to fill in various questionnaires at various time-points before and after the TSST challenge.

### Baseline psychological assessment

Trauma-script challenge experiment: After written informed consent was obtained, all patients were asked to answer standardized demographic questions. PTSD symptoms were assessed with the authorized German translation [34] of the Structured Clinical Interview for DSM-IV (SKID-PTSD) and psychiatric comorbidities with the International Classification of Diseases and Related Health Problems, Tenth Revision (ICD-10) checklists [35]. The authorized German translation of the Structured Clinical Interview for DSM-IV Dissociative Disorders (SCID-D) [36], which comprises five numerical subscales and has an overall score of 20, was used to assess the presence of a dissociative disease. A SCID-D total score of ≥10 reflects a complex and severe dissociative disorder. In addition, self-report data were obtained to evaluate the trauma-related symptom load with the German version of the 15-item self-report Impact of Event Scale (IES) [37] and for dissociative symptoms with the Dissociative Experiences Scale (DES) [38].

TSST challenge experiment: As reported in detail previously [21], medically healthy participants without any present or lifetime psychopathology and without any trauma exposure were identified with the Munich Composite International Diagnostic Interview (M-CIDI) [39] after written informed consent had been obtained.

### Oxytocin treatment and trauma-script challenge of patients

**Fig. 1:**
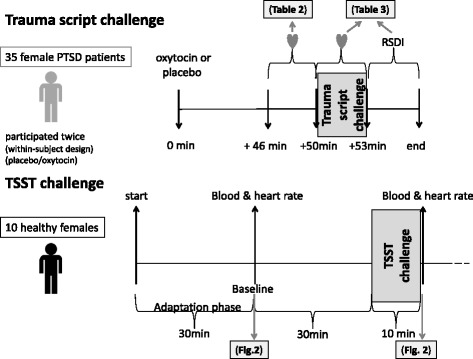
Graphical overview of study design. For further explanations, see “Methods”. The heart symbol represents an assessment of cardiac parameters and respiratory frequency. *diss*. dissociative symptoms, *Abbreviations*: *PTSD* post-traumatic stress disorder, *RSDI* Responses to Script-Driven Imagery Scale, *TSST* Trier Social Stress Test

An overview of both challenge paradigms, that is, of the trauma-script challenge and the TSST challenge paradigm, are depicted in Fig. 1. The study protocol of this RCT (trial registration number: DRKS00009399) is available upon request. The RCT followed the CONSORT guidelines [40]; the CONSORT diagram is provided in Additional file 1: Figure S1 and the CONSORT checklist in Additional file 1: Table S1.

Not later than 14 days after baseline assessments, PTSD patients were subjected to the following challenge protocol: 45 min before start of the stress experiment, PTSD patients received, in a randomized order, either 24 IU oxytocin (Syntocinon®, Novartis, Brazil) or vehicle (placebo) intranasally. The placebo included all of the same ingredients as the oxytocin intranasal spray except oxytocin – inter alia purified water, NaCl and preservatives. Then, the respiratory frequency (RESP) and the cardiovascular parameters HR, HRV and PEP were assessed at baseline, that is, directly before the trauma-script challenge (from minute 46 to minute 50) and again during trauma-script exposure (from minute 51 to minute 53). From minute 53 on, the psychological reaction to stress exposure was assessed with the Responses to Script-Driven Imagery Scale (RSDI) [41]. All patients served as their own controls and were thus assessed twice, that is, they received, in a 1-week interval, oxytocin in one experiment and placebo in the other (within-subject design) in a double-blind randomized order. Patients were randomized using a predefined computer-based block-wise randomization plan performed by a staff member from another institute (the TUM data center). Both the investigators and the patients were blinded to the intervention type until the end of the study. The spray bottles containing either oxytocin or placebo were labeled with a code number by a staff member not otherwise involved in the study. We made every effort to keep blinding integrity, however, we did not assess it. The primary outcome variable was the efficacy of intranasal oxytocin on PTSD symptoms provoked by trauma-script exposure in female PTSD patients. There were no important changes to methods after trial commencement and no drop-outs.

### Psychophysiological assessment upon trauma-script challenge

The script-driven imagery procedure used here differed from the standard approach [42] in that we employed a script of 2 min rather than of 30 s, and we skipped the imagery period at the end of the script [43]. The RSDI [41] was presented to patients as a self-report questionnaire that they filled in in the presence of an investigator. The 11-item RSDI was developed to provide a brief and face valid measure of state PTSD and dissociative symptoms elicited by script-driven imagery, a widely used symptom provocation method in PTSD research. The RSDI measures state re-experiencing, avoidance and dissociative symptoms evoked by script-driven trauma imagery. The predicted three-factor solution is supported by confirmatory factor analyses, including tests for sample invariance across measurement and structural models in three different samples, with the fully constrained model exhibiting good model fit. The response format is a 7-point Likert scale. In the current study, the RSDI was administered in a questionnaire form in the presence of the investigator to ensure comprehension of the directions and to allow participants to ask for clarification about particular items.

Further details on the procedure and devices used for assessment of cardiovascular parameters and of RESP are given in Additional file 1: Supplemental Methods.

### Trier Social Stress Test of healthy participants

The TSST challenge experiment was not part of the RCT but an additional experiment. As explained above, the healthy subjects presented here constitute a subsample of a previously published cohort [21]. Here, we assessed, for the first time, their oxytocin serum levels at baseline and immediately after exposure to the TSST, a standardized social stress experiment [44], the protocol of which we described in detail before [21]. Collection of blood samples and assessment of HR was also described previously [21] and accomplished 30 min before (baseline) and immediately after the TSST, that is, at 1:30 p.m. and at 2:10 p.m. Further details on the procedure of sample processing are given in Additional file 1: Supplemental Methods. We extracted raw HR data for these 10 individuals from our previous publication [21] to correlate with their oxytocin serum levels. As we explained previously [21], subjective stress perception was assessed with the Visual Analogue Scale.

### Enzyme-linked immunosorbent assay analysis of oxytocin serum levels

We used 50 μl of serum from healthy control participants to assess oxytocin serum levels using a commercially available ELISA kit (USCN Life Sciences, Wuhan, China; range of detection: 2.35–1000 pg/ml; sensitivity 4.45 pg/ml) as described by the manufacturer. Samples were assessed in duplicate. Further details on oxytocin analysis are given in Additional file 1: Supplemental Methods.

### Determination of serum cortisol

As we described in further detail previously [21], the concentration of cortisol levels in the serum of healthy subjects was determined with an electrochemiluminescence immunoassay employing the modular analytics EVO analyzer (Roche, Mannheim, Germany).

### Statistics

Data were analyzed using SPSS statistical package version 22. All data were checked for normal distribution.

Trauma-script challenge: Assuming an effect size of 0.5, we calculated a sample size of 34 prior to the study [Input: tail(s) = 2; effect size dz = 0.5; α error probability = 0.05; power (1 − β error probability) = 0.80. Output: non-centrality parameter δ = 2.9154759; critical t = 2.0345153; degrees of freedom = 33; total sample size = 34; actual power = 0.8077775]. Mean values of all psychophysiological data recorded at baseline (during the last 60 s of the 5 min baseline protocol) and when listening to the audiotaped trauma script (first 60 s of the challenge protocol) were calculated. To evaluate physiological data and subjective reactions to the trauma script (RSDI questionnaire), we performed a linear mixed model analysis (LMM) with placebo/oxytocin as the repeated factor (see “Results” and Additional file 1: Table S2). In case of a non-normal distribution of the data, non-parametric tests (Mann–Whitney tests) were conducted. Since the latter results did not differ from the parametric analysis, we report only the results from the LMM. All patients included in this RCT were also included in analyses.

**Fig. 2:**
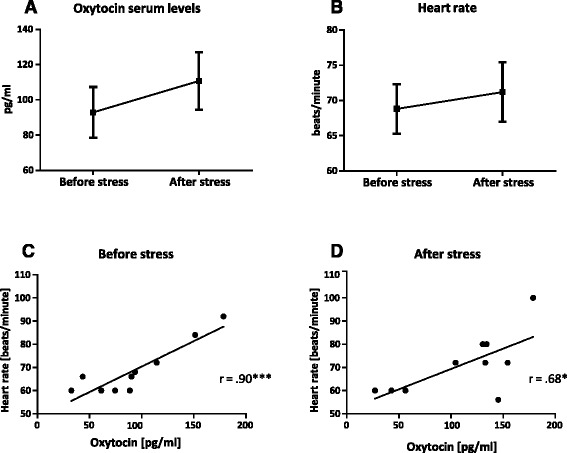
Oxytocin serum levels correlate positively with heart rate both before and after social stress exposure. Ten healthy female participants were subjected to the Trier Social stress Test (TSST). Their serum oxytocin levels and heart rate were assessed at baseline (30 min before) and immediately after the TSST challenge. **a**, **b** Differences in oxytocin serum levels and heart rate before and after stress induction were calculated with separate one-way ANOVAs. **c**, **d** Correlation analyses were performed using Pearson’s correlation coefficient. All data are presented as mean ± standard error of the mean. * *p* ≤ 0.05; *** *p* ≤ 0.001. For further statistical details, see main text

TSST challenge: According to the results of the Kolmogorov–Smirnov test, all data were normally distributed. Differences in oxytocin serum levels, HR, cortisol and subjectively perceived stress before and after stress exposure were calculated with separate one-way ANOVAs (Fig. 2a, b and Additional file 1: Figure S2). The relationship between oxytocin levels and HR before and after TSST exposure was analyzed with Pearson’s correlation coefficient (Fig. 2c, d). The effects of control variables (age, body mass index (BMI), ovarian cycle of healthy participants), all assessed as detailed previously [21], were calculated with partial correlations.

## Results

### Demographics and clinical characteristics of the PTSD patient sample

Demographic and clinical characteristics of this patient cohort are summarized in Table 1. The mean age of the 35 white Caucasian female PTSD patients included was 39.8 years (range 22–70 years). Seventeen of them (49%) had a university-entrance diploma (German: *Abitur*), ten (29%) had a General Certificate of Secondary Education (German: *Realschulabschluss*) and eight (22%) had a Lower General Certificate of Secondary Education (German: *Hauptschulabschluss*). Twenty patients (57%) were in a relationship, 15 (43%) were not. Twenty-one of the study participants (60%) were not married, eight (23%) were married, five (14%) were divorced and one (3%) was widowed. Twelve participants (34%) had children. Eight patients (23%) were full-time employed, eight (23%) were part-time employed, 14 (40%) were retired and five (14%) were unemployed.

**Table 1:** Sociodemographic and clinical characteristics of the post-traumatic stress disorder patient sample subjected to the trauma-script challenge

Characteristic	Mean (SD) or N (%)
Age (years)	39.8 (11.2)
Age (years) at first traumatization	7.7. (4.8)
Female sex	35 (100%)
Education	
< 10 years	8 (22%)
10–12 years	10 (29%)
> 13 years	17 (49%)
Married	8 (23%)
Employment status	
Employed	16 (46%)
Retired	14 (40%)
Unemployed	5 (14%)
Multiple traumatization	29 (83%)
Psychological variables	
SCID-D score	12.1 (4.7)
DES	23.4 (15.7)
IES	46.6 (14.7)

*Abbreviations: DES* Dissociative Experiences Scale, *IES* Impact of Event Scale, *SCID-D* Structured Clinical Interview for Diagnostic and Statistical Manual of Mental Disorders, 4th edition – Dissociative Disorders. For citations, see main article

Eighteen patients (51.4%) received serotonin reuptake inhibitors, eleven patients (31.4%) were treated with antidepressants, nine patients (25.7%) with neuroleptics, five patients (14.3%) with benzodiazepines, twelve patients (34.3%) underwent combined pharmacotherapy and ten patients (28,5%) received no medication. All patients fulfilled DSM-IV diagnostic criteria for PTSD, and PTSD symptoms were their major complaint. The mean age at first traumatization was 7.7 years (range 2–22 years). Most patients (*n* = 26, 74%) suffered from early relational trauma. With regards trauma history, 17 (48.6%) were exposed to sexual violence by a family member, six (17.1%) to non-sexual violence by a family member, four (11.4%) to an accident, three (8.6%) to organized sexual violence, two (5.7%) to sexual violence by a stranger, two (5.7%) to non-sexual violence by a stranger and one (2.9%) to a natural disaster. Thus, 22 (62.9%) of our patients reported a sexual trauma. Because we observed that severe distress and even dissociative symptoms can be provoked by the assessment of sexual functions in sexually traumatized patients, we dispensed with it. With regards comorbidities, 19 participants (54.3%) were diagnosed with a dissociative disorder. Accordingly, the mean SCID-D score of the cohort was >10, namely 12.7 (Table 1). Moreover, 23 patients (65.7%) had a current depressive disorder, 11 (31.4%) an anxiety disorder, ten (28.6%) a somatoform disorder and six (17.1%) a borderline-personality disorder.

### Intranasal oxytocin reduces provoked PTSD symptoms

First, we analyzed the efficacy of oxytocin treatment on PTSD symptoms triggered by trauma-script exposure. Provoked PTSD symptoms were assessed with the RSDI questionnaire that allows quantification of avoidance, re-experiencing and dissociation symptoms provoked by exposure to an audiotaped individual trauma script [41]. We found that the total RSDI score was significantly reduced in oxytocin-treated patients (Table 2, *p* = 0.012). Thus, intranasal oxytocin treatment significantly attenuated PTSD symptoms triggered by trauma-script exposure. Analysis of the three different symptom clusters revealed that the oxytocin-mediated reduction in dissociative and re-experiencing symptoms was not significant (Table 2). However, oxytocin treatment attenuated avoidance – at least with a trend for statistical significance (Table 2, *p* = 0.093). Comparative analysis of the psychophysiological variables of the first and the second experiment revealed no adaptation effects.

**Table 2:** Effects of oxytocin versus placebo on cardiac parameters and trauma-related psychopathological symptoms at baseline and in response to trauma-script challenge

	Placebo		Oxytocin		Repeated effects comparison (LMM)		Effect size
	baseline	Trauma script	baseline	Trauma script			
	mean (SD)	mean (SD)	mean (SD)	mean (SD)	F (DF)	*p*-value	Cohen’s d (95% CI)
HR (bpm)	75.9 (9.6)	82.1 (11.4)	77.9 (8.9)	87.2 (15.0)	3.0 (34)	0.059^t^	0.291 (−0.00–0.50)
HR max (bpm)		88.1 (12.6)		93.8 (15.2)	12.8 (34)	0.020*	0.605 (0.34–0.87)
HRV (ms)	3.15 (.65)	2.91 (.79)	3.05 (.79)	2.79 (.85)	0.26 (34)	0.67	0.088 (−0.15–0.33)
PEP (ms)	76.0 (12.2)	74.2 (12.9)	74.4 (13.6)	70.0 (17.7)	8.8 (33)	0.007**	0.515 (0.26–0.77)
RESP (bpm)	15.6 (3.0)	17.1 (3.8)	15.1 (3.1)	16.7 (4.2)	0.01 (34)	0.95	0.001 (−0.39–0.41)
RSDI-total		2.87 (1.1)		2.44 (.90)	7.1 (34)	0.012*	0.459 (0.13–0.79)
RSDI-Re-experiencing		3.74 (1.3)		3.30 (1.2)	2.2 (34)	0.15	0.342 (−0.11–0.70)
RSDI-Avoidance		2.52 (1.8)		1.92 (1.4)	3.0 (34)	0.093^t^	0.289 (−0.14–0.72)
RSDI-Dissociation		2.26 (1.8)		1.96 (1.4)	1.2 (34)	0.26	0.195 (−0.13–0.52)

*Abbreviations: bpm* beats per minute, *CI* confidence interval. *HR* heart rate, *HR max* heart rate maximum during trauma-script challenge, *HRV* heart rate variability (logarithmically transformed), *LMM* linear mixed model analysis, *PEP* pre-ejection period, *RESP* respiration rate (breaths per minute), *RSDI* Response to Script-Driven Imagery Scale (for citation, see main text), *SD* standard deviation. Symbols: t ≤ 0.1; *p ≤ 0.05; **p ≤ 0.01. All effects of physiological data have been controlled for influences of baseline values

### Intranasal oxytocin exerts positive chronotropic effects

Data presented in Additional file 1: Table S2 are also presented in Table 2; however, in contrast to Table 2, Additional file 1: Table S2 allows for immediate judgment of the statistical significance of the effects of oxytocin treatment on baseline parameters: intranasal oxytocin treatment elicited a significant elevation in the baseline HR of PTSD patients (Additional file 1: Table S2, *p* = 0.02) but no significant differences in the other psychophysiological variables tested at baseline (HRV, PEP, RESP) (Additional file 1: Table S2). The positive chronotropic effect of oxytocin also became apparent during trauma-script exposure because oxytocin-treated patients exhibited a significantly higher maximum HR (Table 2, *p* = 0.020) and, in addition, a trend-wise increase in HR after trauma-script challenge (Table 2, *p* = 0.59). The latter finding indicates that oxytocin treatment promotes the stress-induced increase in HR in PTSD patients.

Because reports on the influence of oxytocin on the regulation of HR are hitherto inconsistent, we analyzed the HR and endogenous oxytocin levels in another cohort, namely a small cohort of healthy women subjected to a different stressor, the standardized social stressor TSST. This cohort constitutes a subsample of a previously published sample [21]. The mean age of the 10 white Caucasian healthy non-traumatized unmedicated participants was 36.08 years (SD = 8.56, range 22–51 years). According to the participants’ self-reports on their first day of the last menstrual period and the average ovarian cycle duration, six participants were in the follicular phase, three in the luteal phase and one reported menopause. Their BMI values were all normal (mean = 23.54 kg/m^2^, SD = 3.13, range 18.07–28.34 kg/m^2^).

As expected from the already published analysis of the total sample [21], TSST exposure of the healthy control subjects tested here induced a substantial increase in their serum cortisol levels (Additional file 1: Figure S2A, F(1, 19) = 24.89, *p* < 0.001) and in the intensity of their subjectively perceived stress (Additional file 1: Figure S2B, (F(1, 19) = 37.40, *p* < 0.001). In contrast, the increase in HR and in oxytocin serum levels was not statistically significant (Fig. 2a, b). However, we found a strong and significant positive correlation between the two parameters both before and after TSST challenge. We cannot exclude that this notably strong correlation results from a methodological artifact driven by the small sample size; however, it did not change significantly after controlling for age (before stress: *r* = 0.93, *p* < 0.001; after stress: *r =* 0.71, *p* = 0.031), BMI (before stress: *r* = 0.91, *p* = 0.001; after stress: *r* = 0.70, *p* = 0.03) and ovarian cycle (before stress: *r* = 0.90, *p* = 0.001; after stress: *r* = 0.67, *p* = 0.048) (Fig. 2c, d). In summary, our data provide strong evidence that oxytocin exerts positive chronotropic effects in women.

### Evidence for sympathomimetic effects of intranasal oxytocin

In order to get an idea of the influence of intranasal oxytocin on the sympathetic and parasympathetic nervous system (SNS, PNS), we assessed additional cardiac parameters. Our finding of a reduction in PEP, a well-known marker of sympathetic cardiac control [32], in oxytocin-treated patients (Table 2, *p* = 0.007) supports the hypothesis that the oxytocin-elicited elevation in HR might be mediated through SNS activation – at least under conditions of stress. In contrast, we found no evidence that oxytocin treatment influenced the parasympathetic tone because the HRV did not differ between oxytocin- and placebo-treated patients – neither at baseline (Additional file 1: Table S2) nor after the trauma-script challenge (Table 2). Taken together, intranasal oxytocin treatment attenuates the intensity of provoked PTSD symptoms (Table 2) despite exerting sympathomimetic and positive chronotropic effects (Additional file 1: Table S2, Table 2). PTSD patients analyzed here did not report unintended effects. Accordingly, besides the positive chronotropic effect of oxytocin treatment, we did not observe any other unintended effect or harm of treatment.

## Discussion

This is the first study assessing the effects of oxytocin on the intensity of provoked PTSD symptoms in female PTSD patients and, to the best of our knowledge, the second symptom provocation study analyzing the efficacy of oxytocin in PTSD patients ever. Taken together, we show here for the first time that intranasal oxytocin reduces the trauma script-provoked expression of PTSD symptoms, in particular avoidance (Table 2). Furthermore, the oxytocin-mediated increase in HR (Additional file 1: Table S2; placebo baseline: 75.9 bpm (SD 9.6); oxytocin challenge: 77.9 bpm (SD 8.9); *p* = 0.020) gets slightly more pronounced in response to stress (Table 2; oxytocin baseline: 82.1 bpm (SD 11.4); oxytocin challenge: 87.2 bpm (SD 15.0); *p* = 0.095). The here-observed immediate influence of intranasal oxytocin on psychopathology and hence on brain function is supported by primate studies showing that intranasal oxytocin, such as other intranasally administered anxiolytic neuropeptides like NPS [45], are able to reach the cerebrospinal fluid [46, 47] and thus, assumingly, also the brain. In addition, our experiments revealed that oxytocin significantly increased the HR in female PTSD patients both at baseline (Additional file 1: Table S2) and upon trauma-script exposure (Table 2), helping to clarify the hitherto still not fully elucidated influence of oxytocin on HR [10, 24–26, 31, 32]. Our finding of a positive chronotropic effect of oxytocin is supported by the positive correlation of endogenous oxytocin levels and HR both before (Fig. 2c) and after (Fig. 2d) TSST challenge in a cohort of healthy subjects.

In contrast to our findings (Fig. 2a, b), de Jong and colleagues found a significant increase in salivary oxytocin levels of healthy subjects in response to TSST exposure [48]. However, from the fact that a correlation of salivary and plasma oxytocin levels was absent in another study on healthy individuals [49], we conclude that our results do not contradict those of de Jong and colleagues. Because there is some controversy on the measurement of oxytocin in the literature, in particular on the determination of oxytocin in unextracted plasma samples [50, 51], we decided to determine oxytocin levels in serum samples and to use a hitherto uncriticized ELISA kit, although it has not yet been extensively validated. In spite of this, the oxytocin levels we report here are higher than those obtained by others with extracted samples. However, the facts that the levels were still much lower than those documented with the criticized assays and, moreover, showed a positive association with stress (Fig. 2c, d), as expected from the extrapolation of previously published data [52], strongly suggest that a potential sample-matrix interference, if existent at all, did not corrupt our results.

Because we found intranasal oxytocin to enhance the challenge-mediated decrease in PEP but not oxytocin-mediated modulation of HRV (Table 2), a known measure for parasympathetic activity [53], we hypothesize that the positive chronotropic effect of oxytocin observed here occurs through an oxytocin-elicited activation of the SNS rather than through an oxytocin-mediated dampening of the PNS. Our results on the influence of oxytocin on HR and PEP in PTSD patients are in full accordance with those of another research group that found intranasal oxytocin to increase the HR and to decrease PEP in healthy individuals [32]. However, in contrast to the latter publication, we did not detect a significant increase in HRV in our cohort (Table 2). This difference might have been caused by differences in study design, but could also indicate that oxytocin-mediated activation of the PNS might be altered in PTSD patients. This supposition is supported by previous studies showing that, besides the SNS [54], the PNS also plays a role in PTSD. For example, parasympathetic activity was found to influence basal HR in PTSD patients [55] and to be lower in Croatian combat veterans [56]. In contrast, it is well accepted that the SNS is overactive in PTSD [54]. Of note, only little of the intranasally administered oxytocin reaches the brain. Accordingly, it has been suggested that the central nervous effects of oxytocin might be a consequence of its peripheral actions [57], such as its influence on cardiac function.

Gender differences in oxytocin effects have been repeatedly described [22, 23] and might, besides a variety of other factors, contribute to the partial differences between our study and that of Norman and colleagues [32], as well as of Pitman and colleagues [42], who, in contrast to us, did not find an effect of intranasal oxytocin on provoked PTSD symptoms in male veterans. In recent years, oxytocin has been repeatedly proposed as an effective novel drug treatment for PTSD [3, 58], but, to the best of our knowledge, there have only been two prior studies that assessed the effects of oxytocin treatment on the intensity of PTSD symptoms in humans [10, 12]. Thus, our study contributes significantly to our understanding of the therapeutic potential of oxytocin in PTSD. The findings of our clinical trial support the use of oxytocin in PTSD treatment, in particular for medication-enhanced exposure therapy. Interestingly, separate analysis of the different PTSD symptoms revealed that avoidance was the only PTSD symptom to be reduced, at least with a trend for statistical significance, by oxytocin treatment (Table 2). Similarly, previous studies found that oxytocin attenuated avoidance behavior in rodents [59, 60]. Because our experiments revealed that oxytocin treatment trend-wise reduced avoidance behavior despite enhancing HR and sympathetic cardiac control (Table 2), we speculate that PTSD-associated avoidance behavior is not causally linked to PTSD-associated SNS overactivity [54].

The relatively small sample size of both samples and the heterogeneity of the patient sample (e.g., the broad range of age, differences in pharmacotherapy and in trauma types) as well as the facts that we analyzed women only and did not include the ovarian cycle phase as a covariate in the analysis of the patient sample limit the generalizability of our results. In a future study we aim to address the question of whether the therapeutic effect of intranasal oxytocin on provoked PTSD symptoms observed here is gender dependent. Moreover, even though we did not observe any unintended effects of oxytocin treatment in addition to its positive chronotropic effects, there is still the possibility of unknown unintended long-term consequences. Future experiments will have to clarify whether the beneficial effects of oxytocin in PTSD treatment described here (Table 2) outweigh the fact that oxytocin stimulates both HR and sympathetic activity (Additional file 1: Tables S2, S3 and [32]), which are both known to be already enhanced in PTSD [54].

## Conclusions

This study provides the first evidence that oxytocin treatment reduces the intensity of provoked PTSD symptoms in female PTSD patients therewith suggesting intranasal oxytocin as a potential novel treatment option for PTSD - in particular for medication-enhanced psychotherapy. Future studies have to explore the gender dependency and the tolerability of the oxytocin-mediated increase in SNS activity and in heart rate.

## Additional file

Additional file 1: Supplemental methods, figures and tables. (PDF 841 kb)

## References

[1] Laine J (1970). Experience of the use of intranasal, buccal and intravenous oxytocin as methods of inducing labour. Acta Obstet Gynecol Scand..

[2] Matsuzaki M, Matsushita H, Tomizawa K, Matsui H (2012). Oxytocin: a therapeutic target for mental disorders. J Physiol Sci..

[3] Koch SBJ, van Zuiden M, Nawijn L, Frijling JL, Veltman DJ, Olff M (2014). Intranasal oxytocin as strategy for medication-enhanced psychotherapy of PTSD: salience processing and fear inhibition processes. Psychoneuroendocrinology..

[4] Gimpl G, Fahrenholz F (2001). The oxytocin receptor system: structure, function, and regulation. Physiol Rev..

[5] Boccia ML, Petrusz P, Suzuki K, Marson L, Pedersen CA (2013). Immunohistochemical localization of oxytocin receptors in human brain. Neuroscience..

[6] Schmidt U, Kaltwasser SF, Wotjak CT (2013). Biomarkers in posttraumatic stress disorder: overview and implications for future research. Dis Markers..

[7] Berger W, Mendlowicz MV, Marques-Portella C, Kinrys G, Fontenelle LF, Marmar CR (2009). Pharmacologic alternatives to antidepressants in posttraumatic stress disorder: a systematic review. Prog Neuropsychopharmacol Biol Psychiatry..

[8] Dine J, Ionescu IA, Avrabos C, Yen Y-C, Holsboer F, Landgraf R (2015). Intranasally applied neuropeptide S shifts a high-anxiety electrophysiological endophenotype in the ventral hippocampus towards a “normal”-anxiety one. PLoS One..

[9] Lin E-J (2012). Neuropeptides as therapeutic targets in anxiety disorders. Curr Pharm Des..

[10] Pitman RK, Orr SP, Lasko NB (1993). Effects of intranasal vasopressin and oxytocin on physiologic responding during personal combat imagery in Vietnam veterans with posttraumatic stress disorder. Psychiatry Res..

[11] Bakermans-Kranenburg MJ, van Ijzendoorn MH (2013). Sniffing around oxytocin: review and meta-analyses of trials in healthy and clinical groups with implications for pharmacotherapy. Transl Psychiatry..

[12] Yazker U, Klein E (2010). Intranasal oxytocin in patients with post traumatic stress disorder: a single dose, pilot double blind crossover study. Eur Neuropsychopharmacol..

[13] Olff M, Koch SB, Nawijn L, Frijling JL, Van Zuiden M, Veltman DJ (2014). Social support, oxytocin and PTSD. Eur J Psychotraumatology..

[14] Acheson D, Feifel D, de Wilde S, McKinney R, Lohr J, Risbrough V (2013). The effect of intranasal oxytocin treatment on conditioned fear extinction and recall in a healthy human sample. Psychopharmacology (Berl).

[15] Frijling JL, van Zuiden M, Koch SBJ, Nawijn L, Goslings JC, Luitse JS (2014). Efficacy of oxytocin administration early after psychotrauma in preventing the development of PTSD: study protocol of a randomized controlled trial. BMC Psychiatry..

[16] Frijling JL, van Zuiden M, Koch SBJ, Nawijn L, Veltman DJ, Olff M (2016). Intranasal oxytocin affects amygdala functional connectivity after trauma script-driven imagery in distressed recently trauma-exposed individuals. Neuropsychopharmacology..

[17] Simeon D, Bartz J, Hamilton H, Crystal S, Braun A, Ketay S (2011). Oxytocin administration attenuates stress reactivity in borderline personality disorder: a pilot study. Psychoneuroendocrinology..

[18] Sack M, Sachsse U, Overkamp B, Dulz B (2013). Trauma-related disorders in patients with borderline personality disorders. Results of a multicenter study. Nervenarzt..

[19] Cardoso C, Ellenbogen MA, Orlando MA, Bacon SL, Joober R (2013). Intranasal oxytocin attenuates the cortisol response to physical stress: a dose-response study. Psychoneuroendocrinology..

[20] Heinrichs M, Baumgartner T, Kirschbaum C, Ehlert U (2003). Social support and oxytocin interact to suppress cortisol and subjective responses to psychosocial stress. Biol Psychiatry..

[21] Zaba M, Kirmeier T, Ionescu IA, Wollweber B, Buell DR, Gall-Kleebach DJ (2015). Identification and characterization of HPA-axis reactivity endophenotypes in a cohort of female PTSD patients. Psychoneuroendocrinology..

[22] Hoge EA, Anderson E, Lawson EA, Bui E, Fischer LE, Khadge SD (2014). Gender moderates the effect of oxytocin on social judgments. Hum Psychopharmacol..

[23] Nishi D, Hashimoto K, Noguchi H, Kim Y, Matsuoka Y (2015). Serum oxytocin, posttraumatic coping and C-reactive protein in motor vehicle accident survivors by gender. Neuropsychobiology..

[24] Gutkowska J, Jankowski M (2012). Oxytocin revisited: its role in cardiovascular regulation. J Neuroendocrinol..

[25] Brotánek V, Kazda S (1965). Differences in the vasodepressor reaction to oxytocin in men and nonpregnant and pregnant women. Am J Obstet Gynecol..

[26] Butwick AJ, Coleman L, Cohen SE, Riley ET, Carvalho B (2010). Minimum effective bolus dose of oxytocin during elective Caesarean delivery. Br J Anaesth..

[27] Ho JM, Blevins JE (2013). Coming full circle: contributions of central and peripheral oxytocin actions to energy balance. Endocrinology..

[28] Yoshida M, Takayanagi Y, Inoue K, Kimura T, Young LJ, Onaka T (2009). Evidence that oxytocin exerts anxiolytic effects via oxytocin receptor expressed in serotonergic neurons in mice. J Neurosci..

[29] Yashpal K, Gauthier S, Henry JL (1987). Oxytocin administered intrathecally preferentially increases heart rate rather than arterial pressure in the rat. J Auton Nerv Syst..

[30] Nakano J, Fisher RD (1963). Studies on the cardiovascular effects of synthetic oxytocin. J Pharmacol Exp Ther..

[31] MacDonald E, Dadds MR, Brennan JL, Williams K, Levy F, Cauchi AJ (2011). A review of safety, side-effects and subjective reactions to intranasal oxytocin in human research. Psychoneuroendocrinology..

[32] Norman GJ, Cacioppo JT, Morris JS, Malarkey WB, Berntson GG, Devries AC (2011). Oxytocin increases autonomic cardiac control: moderation by loneliness. Biol Psychol..

[33] Burg MM, Soufer R (2016). Post-traumatic stress disorder and cardiovascular disease. Curr Cardiol Rep..

[34] Wittchen HU, Zaudig M, Frydrich T (1997). SKID Strukturiertes Klinisches Interview für DSM-IV Achse I und II, Deutsche Version [SCID structured clinical interview for DSM-IV Axis I and II, German Version].

[35] Janca A, Hiller W (1996). ICD-10 checklists—A tool for clinicians’ use of the ICD-10 classification of mental and behavioral disorders. Compr Psychiatry..

[36] Gast U, Oswald T, Zundorf F (2000). Structured Clinical Interview for DSM–IV Dissociative Disorders (SCID-D).

[37] Ferring D, Filipp SH. Teststatistische Überprüfung der Impact of Event-Skala: Befunde zu Reliabilität und Stabilität. Focus Diagnostica. 1994;40:344-362.

[38] Bernstein EM, Putnam FW (1986). Development, reliability, and validity of a dissociation scale. J Nerv Ment Dis..

[39] Wittchen H-U, Pfister H. DIA-X-Interviews: Manual für Screening-Verfahren und Interview. Interviewheft. Frankfurt, Germany: Swets & Zeitlinger; 1997.

[40] Moher D, Hopewell S, Schulz KF, Montori V, Gotzsche PC, Devereaux PJ (2010). CONSORT 2010 Explanation and Elaboration: updated guidelines for reporting parallel group randomised trials. BMJ..

[41] Hopper JW, Frewen PA, Sack M, Lanius RA, Kolk BA (2007). The Responses to Script-Driven Imagery Scale (RSDI): assessment of state posttraumatic symptoms for psychobiological and treatment research. J Psychopathol Behav Assess..

[42] Pitman RK, Orr SP, Forgue DF, de Jong JB, Claiborn JM (1987). Psychophysiologic assessment of posttraumatic stress disorder imagery in Vietnam combat veterans. Arch Gen Psychiatry..

[43] Sack M, Hopper JW, Lamprecht F (2004). Low respiratory sinus arrhythmia and prolonged psychophysiological arousal in posttraumatic stress disorder: heart rate dynamics and individual differences in arousal regulation. Biol Psychiatry..

[44] Kirschbaum C, Pirke K-M, Hellhammer DH (1993). The “Trier Social Stress Test”–a tool for investigating psychobiological stress responses in a laboratory setting. Neuropsychobiology..

[45] Ionescu IA, Dine J, Yen Y-C, Buell DR, Herrmann L, Holsboer F (2012). Intranasally administered neuropeptide S (NPS) exerts anxiolytic effects following internalization into NPS receptor-expressing neurons. Neuropsychopharmacology..

[46] Modi ME, Connor-Stroud F, Landgraf R, Young LJ, Parr LA (2014). Aerosolized oxytocin increases cerebrospinal fluid oxytocin in rhesus macaques. Psychoneuroendocrinology..

[47] Dal Monte O, Noble PL, Turchi J, Cummins A, Averbeck BB (2014). CSF and blood oxytocin concentration changes following intranasal delivery in macaque. PLoS One..

[48] de Jong TR, Menon R, Bludau A, Grund T, Biermeier V, Klampfl SM (2015). Salivary oxytocin concentrations in response to running, sexual self-stimulation, breastfeeding and the TSST: The Regensburg Oxytocin Challenge (ROC) study. Psychoneuroendocrinology..

[49] Javor A, Riedl R, Kindermann H, Brandstätter W, Ransmayr G, Gabriel M (2014). Correlation of plasma and salivary oxytocin in healthy young men—experimental evidence. Neuroendocrinol Lett..

[50] Leng G, Sabatier N. Measuring oxytocin and vasopressin: bioassays, immunoassays and random numbers. J. Neuroendocrinol. 2016;28(10): 10.1111/jne.12413. 10.1111/jne.12413 PMC5096068 27467712

[51] McCullough ME, Churchland PS, Mendez AJ (2013). Problems with measuring peripheral oxytocin: can the data on oxytocin and human behavior be trusted?. Neurosci Biobehav Rev..

[52] Pierrehumbert B, Torrisi R, Laufer D, Halfon O, Ansermet F, Popovic MB (2010). Oxytocin response to an experimental psychosocial challenge in adults exposed to traumatic experiences during childhood or adolescence. Neuroscience..

[53] Hayano J, Sakakibara Y, Yamada A, Yamada M, Mukai S, Fujinami T (1991). Accuracy of assessment of cardiac vagal tone by heart rate variability in normal subjects. Am J Cardiol..

[54] Lipov E (2013). Post traumatic stress disorder (PTSD) as an over activation of sympathetic nervous system: an alternative view. J Trauma Treat..

[55] Hopper JW, Spinazzola J, Simpson WB, van der Kolk BA (2006). Preliminary evidence of parasympathetic influence on basal heart rate in posttraumatic stress disorder. J Psychosom Res..

[56] Jovanovic T, Norrholm SD, Sakoman AJ, Esterajher S, Kozarić-Kovačić D (2009). Altered resting psychophysiology and startle response in Croatian combat veterans with PTSD. Int J Psychophysiol..

[57] Leng G, Ludwig M (2016). Intranasal oxytocin: myths and delusions. Biol Psychiatry..

[58] Olff M, Langeland W, Witteveen A, Denys D (2010). A psychobiological rationale for oxytocin in the treatment of posttraumatic stress disorder. CNS Spectr..

[59] Gaffori OJW, De Wied D (1988). Bimodal effect of oxytocin on avoidance behavior may be caused by the presence of two peptide sequences with opposite action in the same molecule. Eur J Pharmacol..

[60] Kovacs G, Vecsei L, Telegdy G (1978). Opposite action of oxytocin to vasopressin in passive avoidance behavior in rats. Physiol Behav..

