# Supplementary material for: Intranasal oxytocin reduces provoked symptoms in female patients with posttraumatic stress disorder despite exerting sympathomimetic and positive chronotropic effects in a randomized controlled trial
Source: BMC Med. 2017 Feb 17;15:40. doi: 10.1186/s12916-017-0801-0 (PMC5314583; doi:10.1186/s12916-017-0801-0)
Supplement: Additional file 1: — Supplemental methods, figures and tables. (PDF 841 kb) [file 12916_2017_801_MOESM1_ESM.pdf]

SUPPLEMENTAL METHODS

Details on the procedure and devices used for assessment and calculation of cardiovascular parameters

The *peripheral psychophysiological activation* was registered via impedance cardiography using the Ambulatory Monitoring System (AMS; Vrije Universiteit, Department of Psychophysiology, Amsterdam, Netherlands) and seven disposable pre-gelled self-adhesive surface electrodes (Neuroline 720, Ambu, Ballerup, Denmark) attached to the thorax [1].Electrocardiogram (ECG) and impedance cardiography signals (ICG) were recorded. Validity and reliability of the VU-AMS system have been demonstrated [2].

Inter-beat intervals (IBI) were calculated as differences between adjacent R-R signals. Changes in sympathetic drive were measured by changes in the pre-ejection period (PEP). PEP was calculated from the peak of the dZ/dt function of the impedance signal according to the formula given by Lozano et al. [3].

Root mean square of successive differences (RMSSD) of successive inter-beat intervals represented changes in parasympathetic tone. For an estimation of basal parasympathetic cardiac activity, *heart rate variability* (HRV) was analyzed in the time domain. According to published recommendations, RMSSD of five preceding and five following heart beats were specified [4]. Due to the known skewed distribution of the RMSSD, the values were transformed with a natural logarithm for further statistical analyses. Compared to frequency analysis (e.g., Fourier transformation), time series analysis of HRV using RMSSD is known to be of limited power in detecting intraindividual variations of parasympathetic tone [5]. Despite this limitation, RMSSD was chosen as our estimate of parasympathetic drive as this measure is known to be especially useful for detecting short-term variations.

Furthermore, the *band-pass filtered thoracic impedance* allowed an estimation of respiratory frequency. For processing of physiological data the software VU-DAMS 3.2 (Vrije Universiteit, Department of Psychology Amsterdam) was used.

Details on the analysis of serum oxytocin

The detection limit of the used ELISA assay is 12.35 pg/ml with a minimum detectable dose of less than 5.27pg/ml. Furthermore, the manufacturer stated that 20 replications of the analysis of 3 samples on one plate revealed an intra-assay coefficient of variation of <10% and, moreover, that the analysis of 3 samples with low, middle and high oxytocin levels on 3 different plates with 8 replicates in each plate revealed an intra-assay coefficient of variation of <12%. Our analysis revealed an intra-assay coefficient of <7% and an inter-assay coefficient of <8%. None of our measurements was rejected because of poor duplication.

Blood samples for oxytocin analysis were taken at 1330h and 1410h. The samples had a volume of 7,5ml and were immediately processed after clotting. Aliquots of serum (150µl) were stored at -80°C until analysis and were thawed only once, i.e. immediately before ELISA analysis.

Supplemental references

1. de Geus EJ, Willemsen GH, Klaver CH, van Doornen LJ. Ambulatory measurement of respiratory sinus arrhythmia and respiration rate. Biol. Psychol. 1995;41:205–27.

2. Willemsen GH, De Geus EJ, Klaver CH, Van Doornen LJ, Carroll D. Ambulatory monitoring of the impedance cardiogram. Psychophysiology. 1996;33:184–93.

3. Lozano DL, Norman G, Knox D, Wood BL, Miller BD, Emery CF, et al. Where to B in dZ/dt. Psychophysiology. 2007;44:113–9.

4. Camm AJ, Malik M, Bigger JT, Günter B, Cerutti S, Choen R. Task force of the European Society of Cardiology and the North American Society of Pacing and Electrophysiology. Heart rate variability: standards of measurement, physiological interpretation and clinical use. Circulation. 1996;93:1043–65.

5. Berntson GG, Lozano DL, Chen Y-J. Filter properties of root mean square successive difference (RMSSD) for heart rate. Psychophysiology. 2005;42:246–52.

CONSORT Flow Diagram

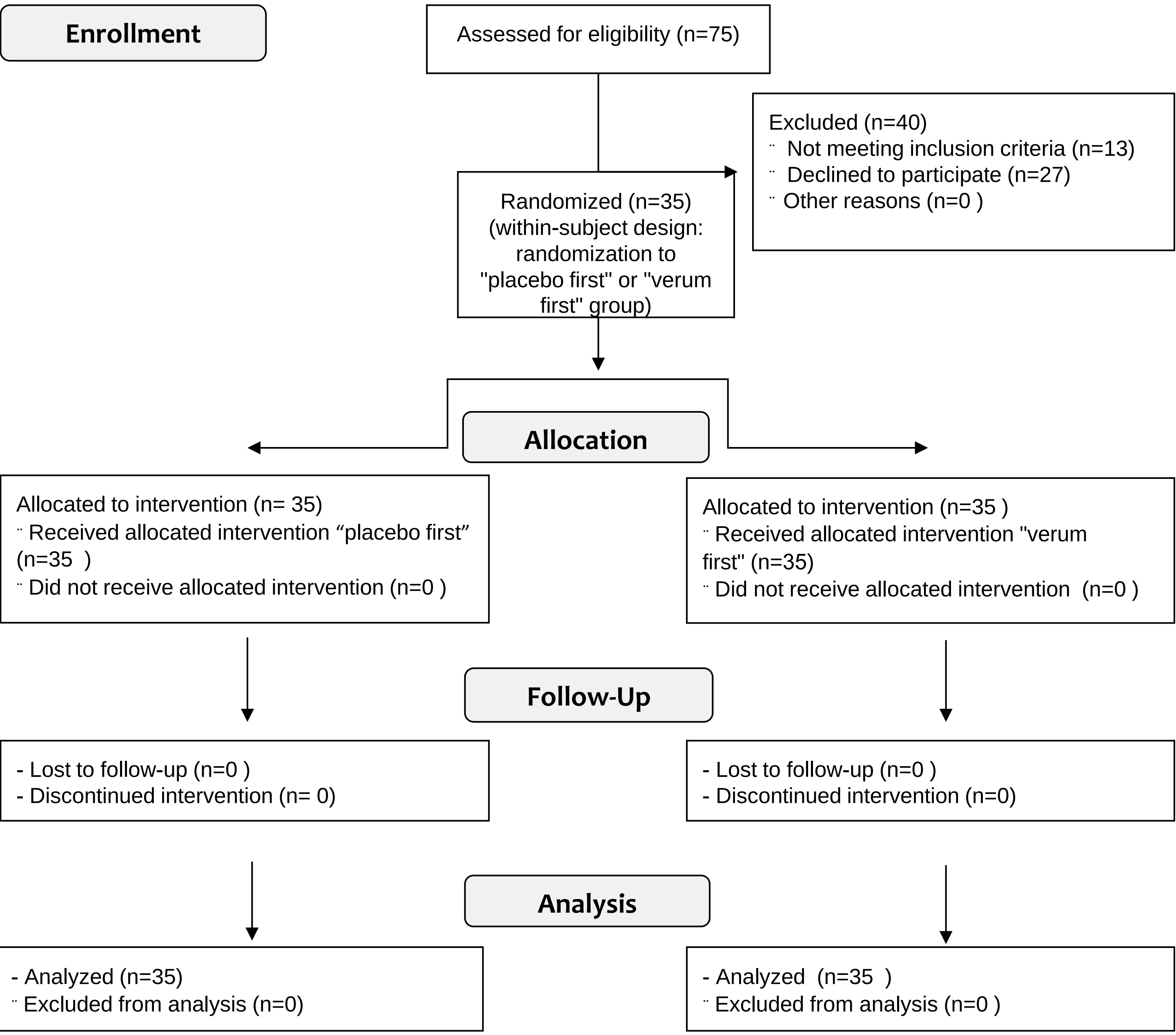

Supplemental Figure 1: Consort Flow Diagram. This diagram was prepared according to the CONSORT guidelines [41]. Note that this randomized controlled trial has a within-subject design, i.e. all participants received both placebo and verum (oxytocin). PTSD patients were randomly assigned either to the "placebo first" or to the "verum first" group. Subjects of the "placebo first" group received first the placebo treatment and one week later the verum treatment while subjects of the "verum group" received treatments in the opposite order. For further details and for references, see main text.

Suppl. Fig. 2

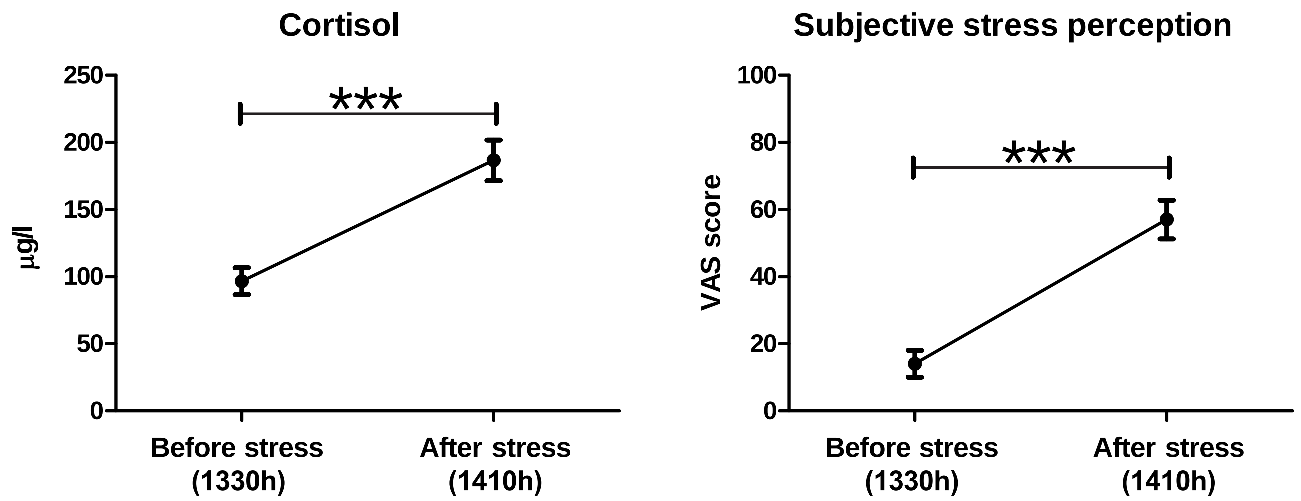

Supplemental Fig.2 Exposure to the Trier Social Stress Test (TSST) induces an increase in serum cortisol levels and in precieved subjective stress in healthy females.

Ten healthy female participants (a subsample of previously published data [21]) were subjected to the TSST. Their serum cortisol levels and their subjective stress response were assessed at baseline, i.e. 30 minutes before, and immediately after the TSST challenge. Differences in cortisol serum levels (**A**) and in subjective stress levels (**B**) were calculated with separate one-way ANOVAs. Inclusion of covariates did not change the results (cortisol: age: $F = 23.93$, $p < .001$; BMI: $F = 36.51$, $p < .001$; ovarian cycle: $F = 23.60$, $p < .001$; subjective stress: age: $F = 44.45$, $p < .001$; BMI: $F = 35.56$, $p < .001$; Ovarian cycle: $F = 43.09$, $p < .001$). Abbreviations: VAS, visual analogue scale. Symbols: *** $p \leq .001$. See main text or further statistical details and references.

SUPPLEMENTAL Table 1: CONSORT checklist for randomised controlled trials

CONSORT 2010 checklist of information to include when reporting a randomised trial*

Section/Topic	Item No	Checklist item	Reported on page No
**Title and abstract**			
	1a	Identification as a randomised trial in the title	1
	1b	Structured summary of trial design, methods, results, and conclusions (for specific guidance see CONSORT for abstracts)	2
**Introduction**			
Background and objectives	2a	Scientific background and explanation of rationale	4-5
	2b	Specific objectives or hypotheses	5-6
**Methods**			
Trial design	3a	Description of trial design (such as parallel, factorial) including allocation ratio	8-11
	3b	Important changes to methods after trial commencement (such as eligibility criteria), with reasons	8 -no changes
Participants	4a	Eligibility criteria for participants	7
	4b	Settings and locations where the data were collected	7
Interventions	5	The interventions for each group with sufficient details to allow replication, including how and when they were actually administered	8-12
Outcomes	6a	Completely defined pre-specified primary and secondary outcome measures, including how and when they were assessed	8
	6b	Any changes to trial outcomes after the trial commenced, with reasons	no changes
Sample size	7a	How sample size was determined	11
	7b	When applicable, explanation of any interim analyses and stopping guidelines	does not apply
Randomisation:			
Sequence generation	8a	Method used to generate the random allocation sequence	9
	8b	Type of randomisation; details of any restriction (such as blocking and block size)	9
Allocation concealment mechanism	9	Mechanism used to implement the random allocation sequence (such as sequentially numbered containers), describing any steps taken to conceal the sequence until interventions were assigned	9
Implementation	10	Who generated the random allocation sequence, who enrolled participants, and who assigned participants to interventions	9

Blinding	11a	If done, who was blinded after assignment to interventions (for example, participants, care providers, those assessing outcomes) and how	9
	11b	If relevant, description of the similarity of interventions	does not apply
Statistical methods	12a	Statistical methods used to compare groups for primary and secondary outcomes	11-12
	12b	Methods for additional analyses, such as subgroup analyses and adjusted analyses	does not apply
**Results**			
Participant flow (a diagram is strongly recommended)	13a	For each group, the numbers of participants who were randomly assigned, received intended treatment, and were analysed for the primary outcome	7 + Fig.1
	13b	For each group, losses and exclusions after randomisation, together with reasons	8 (no exclusions)
Recruitment	14a	Dates defining the periods of recruitment and follow-up	7
	14b	Why the trial ended or was stopped	does not apply
Baseline data	15	A table showing baseline demographic and clinical characteristics for each group	Table 1
Numbers analysed	16	For each group, number of participants (denominator) included in each analysis and whether the analysis was by original assigned groups	10 (all included were analyzed)
Outcomes and estimation	17a	For each primary and secondary outcome, results for each group, and the estimated effect size and its precision (such as 95% confidence interval)	Table 2 and Suppl.Table 2
	17b	For binary outcomes, presentation of both absolute and relative effect sizes is recommended	does not apply
Ancillary analyses	18	Results of any other analyses performed, including subgroup analyses and adjusted analyses, distinguishing pre-specified from exploratory	13 (all exploratory)
Harms	19	All important harms or unintended effects in each group (for specific guidance see CONSORT for harms)	16
**Discussion**			
Limitations	20	Trial limitations, addressing sources of potential bias, imprecision, and, if relevant, multiplicity of analyses	18
Generalisability	21	Generalisability (external validity, applicability) of the trial findings	18
Interpretation	22	Interpretation consistent with results, balancing benefits and harms, and considering other relevant evidence	16-18
**Other information**			
Registration	23	Registration number and name of trial registry	3
Protocol	24	Where the full trial protocol can be accessed, if available	8
Funding	25	Sources of funding and other support (such as supply of drugs), role of funders	22

*We strongly recommend reading this statement in conjunction with the CONSORT 2010 Explanation and Elaboration for important clarifications on all the items. If relevant, we also recommend reading CONSORT extensions for cluster randomised trials, non-inferiority and equivalence trials, non-pharmacological treatments, herbal interventions, and pragmatic trials. Additional extensions are forthcoming: for those and for up to date references relevant to this checklist, see www.consort-statement.org.

SUPPLEMENTAL Table 2: Effects of oxytocin vs. placebo on physiological variables at baseline (before trauma script challenge)

Abbreviations: LMM: linear mixed model analysis; HR, heart rate; HRV, heart rate variability (logarithmically transformed); PEP, pre-ejection period; RESP, respiration rate (breathes per minute); ns, not significant. Symbols: * p ≤ .05.

	**Drug treatment**			
	**Placebo**	**Oxytocin**	**Repeated Effects Comparison (LMM)**	
	**Mean (SD)**	**Mean (SD)**	**F (DF)**	**p**
**HR**	75.4 (9.6)	77.9 (8.9)	6.0 (34)	.020*
**HRV**	3.15 (.65)	3.05 (.62)	2.0 (34)	n.s.
**PEP**	76.0 (12.2)	74.5 (13.8)	2.3 (33)	n.s.
**RESP**	15.6 (3.0)	15.1 (3.2)	2.1 (34)	n.s.
